# The impact of FOXO on dopamine and octopamine metabolism in *Drosophila* under normal and heat stress conditions

**DOI:** 10.1242/bio.022038

**Published:** 2016-10-17

**Authors:** Nataly E. Gruntenko, Natalya V. Adonyeva, Elena V. Burdina, Evgenia K. Karpova, Olga V. Andreenkova, Daniil V. Gladkikh, Yury Y. Ilinsky, Inga Yu Rauschenbach

**Affiliations:** 1 Institute of Cytology and Genetics, Siberian Branch of Russian Academy of Sciences, Novosibirsk 630090, Russia; 2 Institute of Chemical Biology and Fundamental Medicine, Siberian Branch of Russian Academy of Sciences, Novosibirsk 630090, Russia

**Keywords:** FOXO, Insulin signalling, Neurohormonal stress response in insects, *Drosophila*, Dopamine, Octopamine

## Abstract

The forkhead boxO transcription factor (FOXO) is a component of the insulin signalling pathway and plays a role in responding to adverse conditions, such as oxidative stress and starvation. In stressful conditions, FOXO moves from the cytosol to the nucleus where it activates gene expression programmes. Here, we show that FOXO in *Drosophila melanogaster* responds to heat stress as it does to other stressors. The catecholamine signalling pathway is another component of the stress response. In *Drosophila*, dopamine and octopamine levels rise steeply under heat, nutrition and mechanical stresses, which are followed by a decrease in the activity of synthesis enzymes. We demonstrate that the nearly twofold decline of FOXO expression in *foxo^BG01018^* mutants results in dramatic changes in the metabolism of dopamine and octopamine and the overall response to stress. The absence of FOXO increases tyrosine decarboxylase activity, the first enzyme in octopamine synthesis, and decreases the enzymatic activity of enzymes in dopamine synthesis, alkaline phosphatase and tyrosine hydroxylase, in young *Drosophila* females. We identified the juvenile hormone as a mediator of FOXO regulation of catecholamine metabolism. Our findings suggest that FOXO is a possible trigger for endocrinological stress reactions.

## Abstract (precis)

**Summary:** The transcription factor FOXO affects catecholamine metabolism under normal and heat stress conditions in *D. melanogaster*, and juvenile hormone (JH) is a mediator of this effect.

## INTRODUCTION

The endocrinological aspects of the stress response in insects include hormonal and neurotransmitter levels, and these parameters change sharply when an insect faces a stressor, such as adverse temperatures, mechanical and chemical stimuli, high population density, nutrient deprivation or immobilization (Perić-Mataruga et al., 2006; Gruntenko and Rauschenbach, 2008; Even et al., 2012). The biogenic amines, dopamine (DA) and octopamine (OA) are the prominent elements of this response. In *Drosophila melanogaster*, DA has a significant impact on survival during heat stress, starvation (Gruntenko et al., 2004; Ueno et al., 2012) and oxidative stress (Hanna et al., 2015). DA levels rise sharply 30 min after *D. melanogaster* are exposed to stress (38°C) (Gruntenko et al., 2000). The same pattern was observed for DA and OA in *D. virilis* (Rauschenbach et al., 1993; Hirashima et al., 2000). Increased levels of DA and OA were accompanied by a decrease in the activity of the enzyme tyrosine hydroxylase (TH), that catalyses the first and rate-limiting step in DA synthesis (Neckameyer, 1996), continuing for up to 60 min of stress exposure (Rauschenbach et al., 1995). Increased levels of DA and OA are also accompanied by a decrease in the activity of the enzyme alkaline phosphatase (ALP), that regulates the pool of the DA and OA precursor, tyrosine (Wright, 1987), continuing for up to 140 min of stress exposure (Sukhanova et al., 1996; Bogomolova et al., 2010); and a decrease in the activity of the enzyme tyrosine decarboxylase (TDC) (Livingstone and Tempel, 1983), involved in OA synthesis, continuing for up to 120 min of stress exposure (Sukhanova et al., 1997; Gruntenko et al., 2004, 2009; Neckameyer and Weinstein, 2005).

The insulin/insulin-like growth factor signalling pathway (IIS) in *Drosophila* also contributes to stress resistance (Broughton et al., 2005; Söderberg et al., 2011; Rauschenbach et al., 2014a). The *Drosophila* IIS has many components including a transcription factor in the forkhead box class O family (dFOXO), an insulin-like receptor (InR), the fly orthologue of insulin receptor substrates (CHICO), insulin-like peptides, phosphatidylinositol 3-kinase, 3-phosphoinositide-dependent protein kinase 1 and protein kinase B (dAkt1) (Toivonen and Partridge, 2009; Kannan and Fridell, 2013). Cell stress signalling pathways, including oxidative stress and starvation, stimulate dFOXO (Jünger et al., 2003; Hwangbo et al., 2004). Under normal conditions, dFOXO is inactive, inhibited by IIS; in the absence of IIS, in particular during nutrient deprivation, dFOXO translocates to the nucleus where it activates gene expression (Jünger et al., 2003; Puig et al., 2003; Wang et al., 2014). The environmental stressors are also known to cause changes in the expression of hundreds of genes (Lopez-Maury et al., 2008). An alternative mechanism to regulate the activity of FOXO appears to occur through the juvenile hormone (JH) (Mirth et al., 2014). FOXO activity is significantly higher in *D. melanogaster* larvae with genetically ablated *corpora allata*, the organ that produces JH (Mirth et al., 2014). Overexpression of dFOXO increases oxidative stress tolerance (Giannakou et al., 2004; Hwangbo et al., 2004) while dFOXO-null mutants are more sensitive to oxidative stress (Jünger et al., 2003) and deficient in viral defence (Spellberg and Marr, 2015).

However, dFOXO impact on biogenic amines metabolism and dFOXO activity in response to heat stress remain uncharacterized. Here, we examined the effect of a strong hypomorphic mutation *foxo^BG01018^* on DA and OA metabolism in *D. melanogaster* under normal and heat stress conditions. We also studied the effect of heat stress on the cellular location of FOXO protein in the *Drosophila* fat body.

## RESULTS

### Does heat shock affect cellular localization of FOXO?

**Fig. 1.**
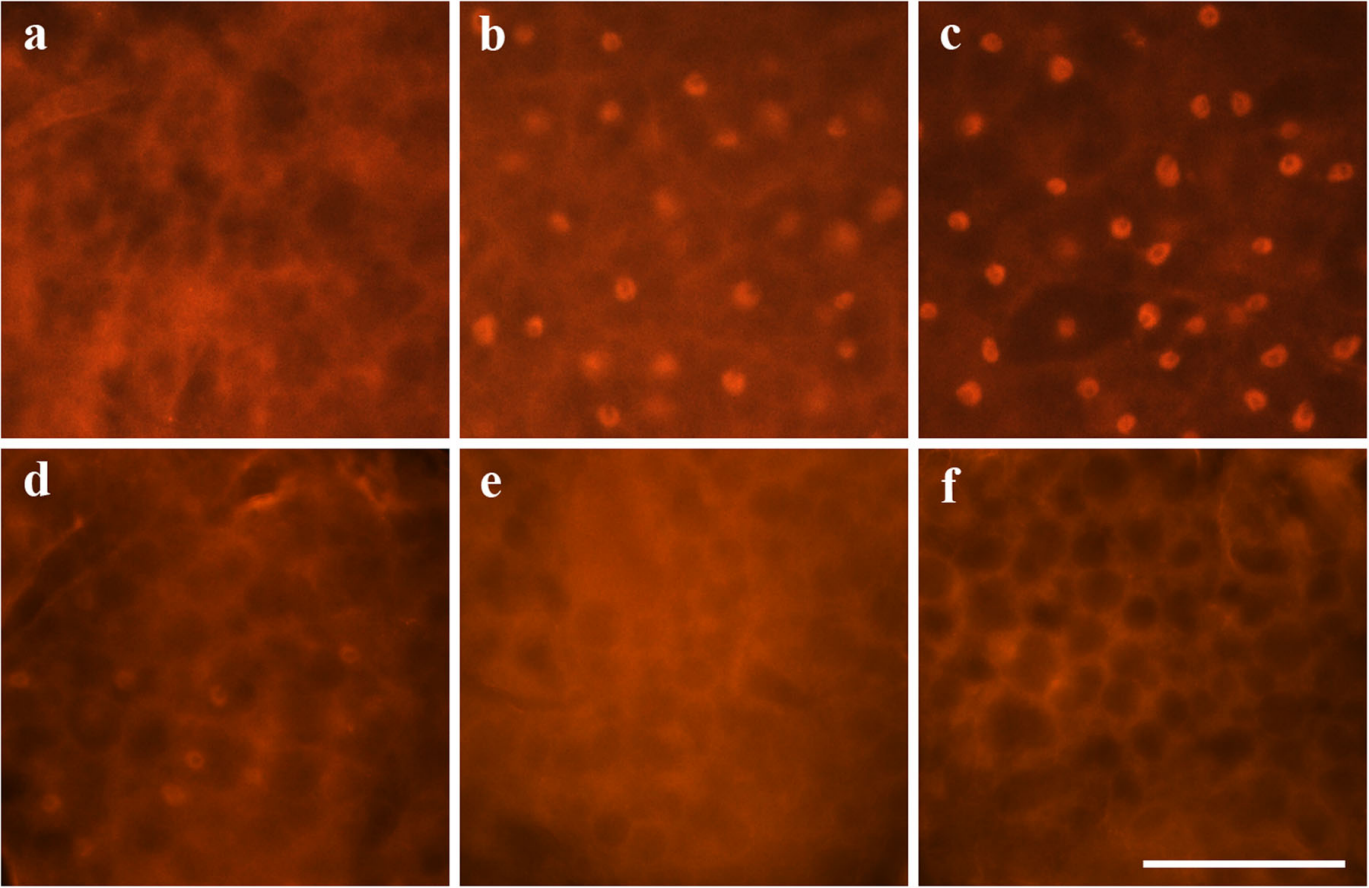
**Cellular localization of dFOXO in the abdominal fat body of six-day-old Canton-S females.** (A) Under normal conditions, (B) after 15 min of heat stress (38°C), (C) after 1 h of heat stress (38°C), (D) 30 min after 1 h of heat stress (38°C), (E) 1 h after 1 h of heat stress (38°C), (F) 1.5 h after 1 h of heat stress (38°C). Scale bar is 50 μm.

To find out whether heat shock, like a decrease in IIS activity, induces dFOXO translocation from the cytoplasm, we examined the cellular location of dFOXO protein during and after heat stress in the fat body of Canton-S females, where dFOXO is predominantly expressed (Zheng et al., 2007). Under normal conditions (Fig. 1A), antibody-labelled dFOXO was distributed throughout the cytoplasm in all cells, and no signal was detected in the nucleus. Fifteen minutes of heat exposure (38°С) increased dFOXO nuclear localization, although the dFOXO protein was still detected in the cytoplasm (Fig. 1B). Following 60 min of heat stress, all detectable dFOXO signals were localized in the nucleus (Fig. 1C). Thirty minutes after the end of heat shock, we observed an increase in the cytoplasmic localization of dFOXO and a decrease in nuclear localization (Fig. 1D). Sixty minutes after heat shock was complete, there was no detectable dFOXO protein in the nucleus while the cytoplasmic signal was at its strongest (Fig. 1E). At 90 min after 1 h of heat stress, the cellular distribution of dFOXO was similar to that in untreated flies (Fig. 1F).

### The *foxo^BG01018^* mutation decreases *dfoxo* transcriptional activity

To study the effect of dFOXO on DA metabolism, we used *D. melanogaster* females with a *foxo^BG01018^* mutation that causes a mild loss of function (Dionne et al., 2006). We evaluated the transcription levels of *dfoxo* and *Tubulin* and found that the *foxo^BG01018^* mutation did not affect the transcription of *Tubulin*, but did cause a decrease in the level of *dfoxo* mRNA, as measured by quantitative RT-PCR. The expression of *dfoxo* in the sample group was down-regulated by a mean factor of 0.564 (s.e. range 0.352–0.846) when compared with the control group (analysis of data by REST 2009); the *dfoxo* sample group differed from the Canton-S control group (*P*=0.012).

### Decreased *dfoxo* expression increases OA synthesis and response of TDC activity to heat stress

**Fig. 2.**
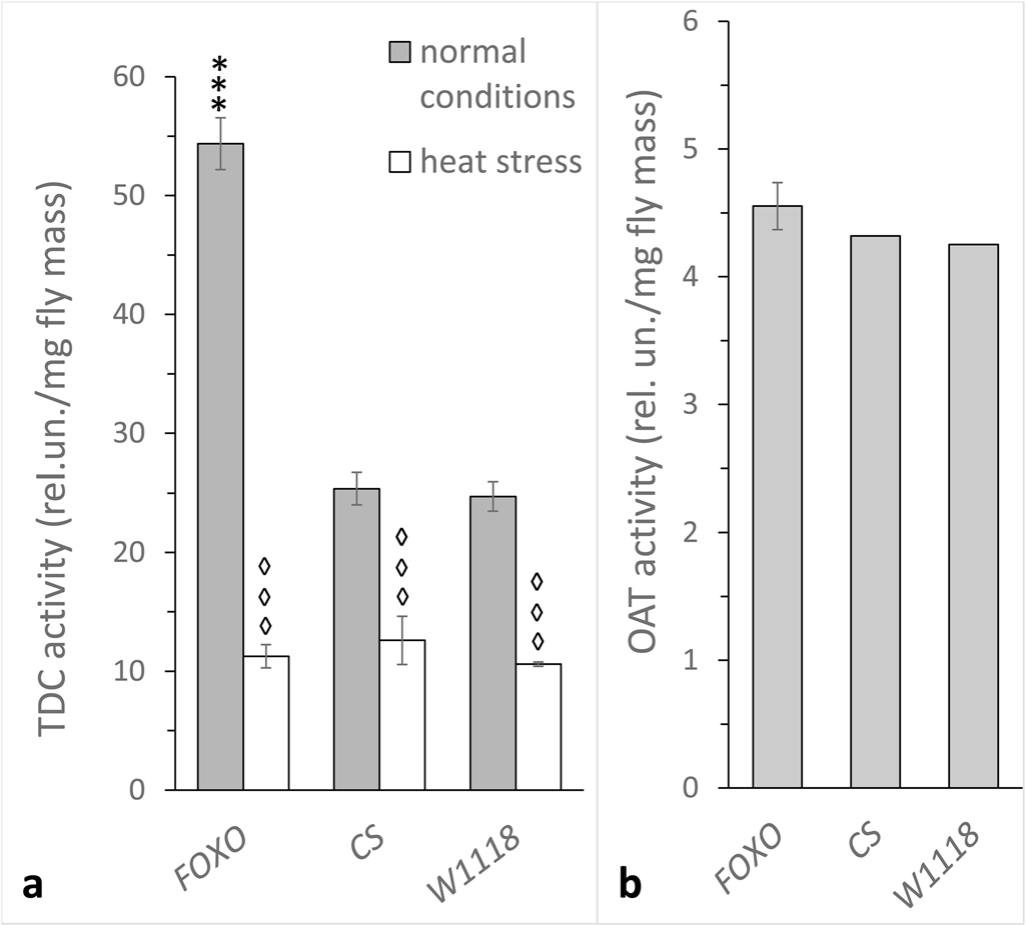
**Effects of decreased *dfoxo* expression on OA metabolism in *D. melanogaster* females.** (A) TDC activity under normal and heat stress conditions (38°C). (B) OAT activity under normal conditions in one-day-old *foxo^BG01018^* (FOXO), Canton-S (CS) and *w^1118^* (W1118) females. Each histogram bar represents an average value of 7 to 8 measurements for TDC and 10 to 12 measurements for OAT. Error bars indicate s.e.m. An asterisk indicates differences between FOXO and control females while a diamond indicates differences between heat-treated and control flies of the same genotype. Three diamonds or asterisks indicates *P*<0.001.

To reveal the possible effect of dFOXO on OA metabolism, we tested whether decreased *dfoxo* expression alters TDC activity (Fig. 2A) under normal and heat stress conditions (38°C, 60 min). Under normal conditions, TDC activity in one-day-old *foxo^BG01018^* females was significantly higher than in the Canton-S and *w^1118^* controls (*P*<0.001). The mutant and control females both responded to heat stress with a decrease in TDC activity; however, TDC activity in the *foxo^BG01018^* females decreased by 79%, which was significantly different from the activity decrease in control groups (52% in Canton-S and 56% in *w^1118^* females; *Р*<0.001).

N-acetyltransferase is not a component of the *Drosophila* stress response (Rauschenbach et al., 1997, 2008), so we measured OA-dependent arylalkylamine N-acetyltransferase (OAT) activity in one-day-old *foxo^BG01018^* females only under normal conditions (Fig. 2B). We found no difference between females with decreased *dfoxo* expression and control groups.

### With a shortage of FOXO, DA metabolism and stress reactivity decrease

**Fig. 3.**
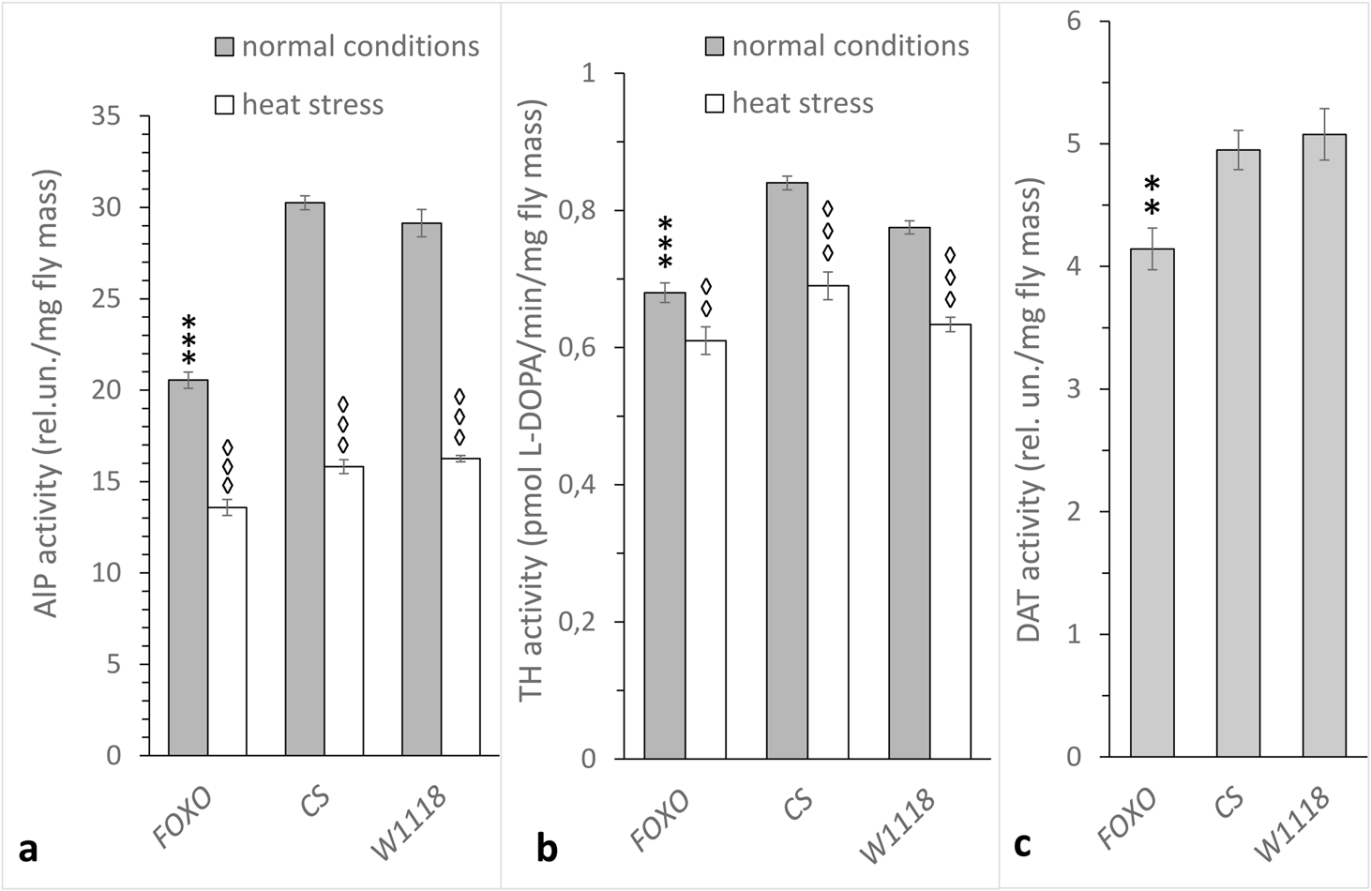
**Effects of decreased *dfoxo* expression on DA metabolism in *D. melanogaster* females.** (A) ALP and (B) TH activities under normal conditions and upon heat stress (38°C), and (C) DAT activity under normal conditions in one-day-old *foxo^BG01018^* (FOXO), Canton-S (CS) and *w^1118^* (W1118). Each histogram bar represents an average of 18 to 20 measurements for ALP, 12 to 18 measurements for TH and 12 to 14 measurements for DAT. Error bars indicate s.e.m. An asterisk indicates differences between FOXO and control females while a diamond indicates differences between heat-treated and control flies of the same genotype. Two diamonds or asterisks indicates *P*<0.01, three diamonds or asterisks indicates *P*<0.001.

The shortage of dFOXO in one-day-old females caused a decline in both the synthesis and degradation of DA (Fig. 3). Under normal conditions, the activity of ALP, TH and DA-dependent arylalkylamine N-acetyltransferase (DAT) in *foxo^BG01018^* females was decreased compared with Canton-S and *w^1118^* controls (*P*<0.001 for ALP and TH, *P*<0.01 for DAT). In addition, the stress reactivity of enzymes in the DA synthesis pathway was reduced. In *foxo^BG01018^* females under heat stress, ALP activity was decreased by 34% compared with controls of 45% in the Canton-S and 44% in *w^1118^* females. The activity of TH was deceased by 10% in *foxo^BG01018^* mutants compared with a decrease of 18% in Canton-S and 19% in *w^1118^* (*Р*<0.001 for both ALP and TH).

These data suggest that DA levels in young dFOXO-deficit females are elevated, consistent with our previous study showing that DA downregulates the activity of its synthesis enzymes (Bogomolova et al., 2010). Low levels of DAT activity may cause an increase in DA levels, which in turn could decrease ALP and TH activity. The decreased DAT activity in *foxo^BG01018^* females may be due to decreased levels of JH. Previous studies have shown that JH metabolism is disrupted in *foxo^BG01018^* females (Rauschenbach et al., 2015) and that JH regulates DA content via DAT in young *Drosophila* females (Rauschenbach et al., 2011, 2014а).

### JH treatment rescues DA and OA synthesis in dFOXO-deficit females

**Fig. 4.**
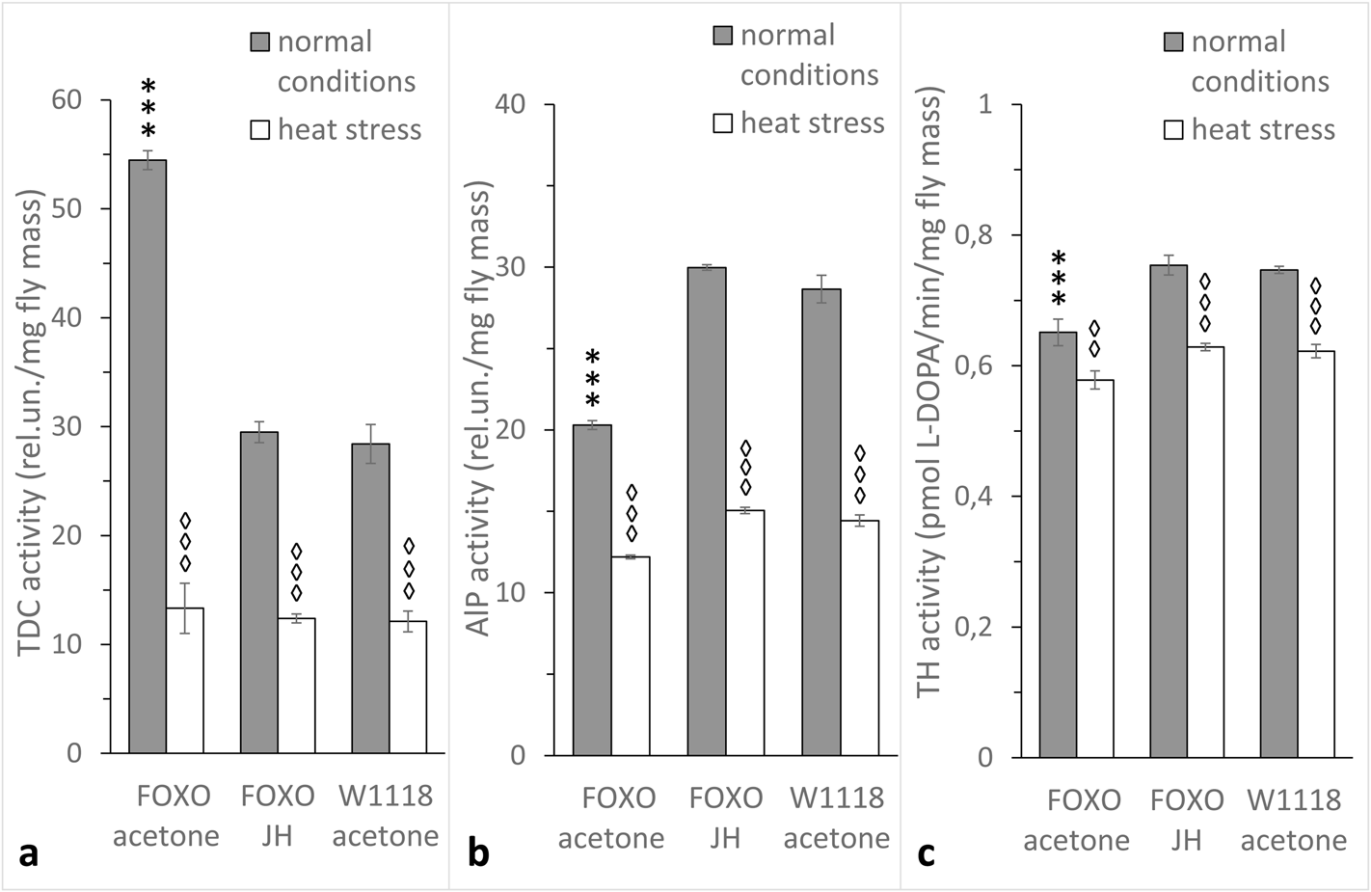
**Effects of JH treatment on TDC, ALP and TH activities under normal conditions and during heat stress (38°C) in one-day-old *foxo^BG01018^* (FOXO) females.** (A) TDC, (B) ALP and (C) TH activities. Control FOXO and W1118 flies were treated with acetone. Each histogram bar represents an average value of 8 to 10 measurements. Error bars indicate s.e.m. The asterisk indicates differences between acetone treated FOXO, JH treated FOXO and acetone treated W1118 females. A diamond indicates differences between heat-treated and control flies of the same group. Two diamonds or asterisks indicates *P*<0.01, three diamonds or asterisks indicates *P*<0.001.

To determine the role of JH in regulating the metabolism of biogenic amines by dFOXO, we investigated the impact of JH treatment on TDC, ALP and TH activities in one-day-old *foxo^BG01018^* females (Fig. 4). For comparison, groups of *foxo^BG01018^* and control *w^1118^* females were treated with pure acetone. Half of the individuals in each group were exposed to heat stress. In *foxo^BG01018^* females treated with JH, TDC activity was decreased, and ALP and TH activities were increased to the level of the control group. The stress reactivity of all three enzymes was also normalized by JH treatment.

## DISCUSSION

Our results provide experimental evidence that the response of dFOXO to stress is universal. Heat shock (Fig. 1), starvation and oxidative stress (Jünger et al., 2003; Hwangbo et al., 2004) cause dFOXO translocation from the cytosol to the nucleus, where it acts as a transcription factor.

Previously, we showed that mutations affecting the signalling pathways of stress-related hormones, such as DA, OA and JH, alter the extent to which other links in the stress response pathway are activated. In particular, the *ebony* mutation, which doubles DA levels (Hodgetts and Konopka, 1973), and the Tβ*h^nM18^* mutation, which causes the total absence of OA (Monastirioti et al., 1996), both decrease the stress reactivity of OA and DA metabolic systems in one-day-old *D. melanogaster* females (Gruntenko et al., 2000, 2004). Here, we report that the *foxo^BG01018^* mutation has similar effects on the stress reactivity of the DA metabolic system (see Results), suggesting that FOXO plays a role in controlling the endocrinological aspects of the stress response. However, TDC stress reactivity in *foxo^BG01018^* females, unlike *ebony* and *T*β*h^nM18^*, is increased compared with wild type (see Results). This may indicate that the impact of dFOXO on the metabolism of biogenic amines is mediated by JH, which upregulates the activity of ALP, TH and DAT, and stress reactivity of ALP and TH, while downregulating the activity and stress reactivity of TDC in young *D. melanogaster* females (Rauschenbach et al., 2007, 2014a,b; Bogomolova et al., 2009; Gruntenko et al., 2012a).

In previous reports, we have shown that JH metabolism is disrupted by the *foxo^BG01018^* mutation (Rauschenbach et al., 2015). Here we report that JH treatment rescues TDC, TH and ALP activity and stress reactivity levels in *foxo^BG01018^* females (see Fig. 4). Thus, we hypothesize that stress triggers the response of dFOXO, which triggers an increase in JH titre, in turn modifying the metabolism of biogenic amines. This hypothesis is consistent with studies investigating whether suppressing InR, the other IIS element, in the *corpus allatum* alters the activity of ALP and TH and their response to heat stress (Rauschenbach et al., 2014a). DAT, ALP and TH activities were decreased in females with reduced InR expression in the *corpus allatum*, and JH treatment restored DA metabolism in these flies (Rauschenbach et al., 2014a) as well as in *foxo^BG01018^* females (see Figs 3 and 4). Consistently, a knockdown of the *InR* gene in the *corpus allatum* had no effect on OAT activity while it increased TDC activity (Rauschenbach et al., 2014b). Altogether, these data support our observations on the effect of the *foxo^BG01018^* mutation on OA metabolism (Fig. 2). It was shown that the environmental stressors cause an increase in the content of OA and DA (Gruntenko and Rauschenbach, 2008). It is also important to note that, under normal conditions, the lack of dFOXO decreases DA metabolism and leads to increased OA metabolism in young *Drosophila* females (Figs 2 and 3).

Kodrík et al. (2015) suggest that FOXO confers oxidative-stress resistance via the transcriptional upregulation of genes encoding anti-oxidative enzymes. Our study suggests that FOXO also influences stress resistance via JH signalling and by regulating DA levels, which affect resistance to heat and nutritional stresses (Gruntenko et al., 2004). This is further supported by the predominant expression dFOXO in the fat body (Zheng et al., 2007), which produces the esterase that degrades JH in insects (Gilbert et al., 2000), and by the decreased survival of *foxo^BG01018^* mutants under heat stress (Rauschenbach et al., 2015).

Altogether, these studies suggest that dFOXO: (1) responds to various stressors by translocating from the cytosol to the nucleus; (2) controls the metabolism of biogenic amines under normal conditions; and (3) control is mediated by the JH signalling system.

## MATERIALS AND METHODS

### *Drosophila melanogaster* stocks and maintenance

In this study, we used *foxo^BG01018^* flies, which carry a P[GT1] element transposon in the 5′ upstream region of the *dfoxo* gene, resulting in a mild loss of function (Dionne et al., 2006). Two different fly lines that are wild-type for the catecholaminergic pathway, *w^1118^* and Canton-S (Hanna et al., 2015) were used as controls. All stocks were obtained from the Bloomington Stock Center (Indiana University, IN, USA) and kept at 25°C, 12 h light:12 h dark photoperiod, in a standard *Drosophila* medium. Flies hatched within 3-4 h were pooled for experiments.

### Heat stress

Flies were exposed to heat stress by transferring vials containing five experimental flies from a 25°C incubator to a 38°C incubator for 1 h (when examining TDC and TH responses to stress), or for 1 h 40 min (when examining ALP responses to stress). The optimum exposure times for each enzyme measurement was determined as previously described (Bogomolova et al., 2010; Gruntenko et al., 2012a).

### Quantitative real-time polymerase chain reaction (qRT-PCR)

Total RNA was extracted from six samples of whole body homogenates of five-day-old Canton-S and *foxo^BG01018^* females (five flies per sample) using TRIzol (Invitrogen, Carlsbad, CA, USA) according to the manufacturer's instructions. Synthesis of cDNA was carried out using dT15 priming and the SuperScript III Reverse Transcriptase (Thermo, Waltham, MA, USA). Expression of *dfoxo* was analysed on an iQ5 (Bio-Rad, Hercules, CA, USA) by qRT-PCR using three replicates for every sample. Data were normalized against Tubulin according to Ponton et al. (2011). The qPCR mix contained BioMaster qPCR SYBR Blue (Biosan, Riga, Latvia), and one of the following primers sets: dFOXOex6-f 5′-GCCTAGATCACTTTCCCGAG-3′, dFOXOex7-r 5′-GTCAGCTCATCCGCCATTGT-3′, Tubulin-f 5′-TGTCGCGTGTGAAACACTTC-3′, Tubulin-r 5′-AGCAGGCGTTTCCAATCTG-3′.

### Immunohistochemistry and fluorescence microscopy

Immunostaining was carried out on six-day-old Canton-S females as previously described (Gruntenko et al., 2012b). The following primary and secondary antibodies were used: rabbit anti-dFOXO polyclonal antibody (1:500, # CAC-THU-A-DFOXO, Cosmo Bio, Tokyo, Japan) and goat anti-rabbit Cy3 (1:400, ab6939, Abcam, Cambridge, UK). Samples were mounted in Vectashield (Vector Laboratories, Burlingame, CA, USA) and imaged using a Zeiss Axioskop 2 Plus microscope.

### Enzyme activity assays

TDC and TH activities were measured in the whole fly homogenate by radioisotope methods as described in Gruntenko et al. (2012a) using a Rackbeta 1209 scintillation counter (Vellag, Turku, Finland). DA- and OA-dependent arylalkylamine N-acetyltransferase (DAT and OAT, respectively) and ALP activity measurements were performed as previously described (Bogomolova et al., 2010; Gruntenko et al., 2012a) with DA (Sigma-Aldrich, St. Louis, MO, USA), OA (Sigma-Aldrich) and α-naphthylphosphate (ICN, Moscow, Russia) as substrates for DAT, OAT and ALP, respectively. The optical density of the obtained reaction products was measured with a SmartSpec™Plus spectrophotometer (Bio-Rad, Philadelphia, PA, USA) at 405 nm (DAT and OAT) and 470 nm (ALP) against the reaction zero point. DAT, OAT and ALP activities are shown as relative units (optical density×100).

### JH treatment

A 1 μg aliquot of JH-III (Sigma-Aldrich) dissolved in 0.5 μl of acetone was applied to the abdomen of one-day-old females. Control females were treated with acetone (0.5 μl). 10 h after application, half of the JH- and acetone-treated flies were exposed to heat stress and then all of the flies were frozen in liquid nitrogen and stored at −20°C.

### Statistics

Data are plotted as the mean±s.e.m. Student's *t*-test was used for all pairwise comparisons of differences in means. Stress-reactivity was calculated as the percentage by which enzyme activity decreased under heat stress: each value obtained at 38°C was compared with the average value obtained at 25°C (Gruntenko et al., 2012a). Statistical analyses were performed in Excel 2013 and REST 2009 (http://www.gene-quantification.de/rest-2009.html).
